# Dental Exostosis

**Published:** 1845-03

**Authors:** 


*Dental Exostosis.*—While on a short visit to Wilmington, N. C., in February, 1844, Dr. Ware, resident dentist of that place, presented us with a superior molar tooth, having on its roots an exostosis the size of a large hickory-nut. It was extracted from the mouth of a negro woman, and had given rise to extensive disease of the maxillary sinus.

We have another equally well marked example of this curious affection, and for which, we are indebted to Drs. Blandin and Reynolds, of Columbia, S. C. It had caused the union of two teeth; a second superior molaris and dens sapientiæ. The deposition of bone, however, in the latter case is not so great as in that of the former.—*Balt. Ed.*

